# Perceived neighbourhood environmental attributes and prospective changes in TV viewing time among older Australian adults

**DOI:** 10.1186/s12966-015-0208-2

**Published:** 2015-04-11

**Authors:** Ai Shibata, Koichiro Oka, Takemi Sugiyama, Ding Ding, Jo Salmon, David W Dunstan, Neville Owen

**Affiliations:** Baker IDI Heart and Diabetes Institute, Level 4, 99 Commercial Rd, Melbourne, VIC 3004 Australia; Faculty of Health and Sport Sciences, University of Tsukuba, 1-1-1 Tennodai, Tsukuba, Ibaraki 305-8577 Japan; Faculty of Sport Sciences, Waseda University, 2-579-14 Mikajima, Tokorozawa, Saitama 359-1192 Japan; School of Design, Swinburne University of Technology, Melbourne, VIC Australia; School of Population Health, University of South Australia, North Terrace, Adelaide, SA 5000 Australia; Sydney Medical School and Sydney School of Public Health, The University of Sydney, Sydney, NSW 2006 Australia; Centre for Physical Activity and Nutrition Research, Deakin University, 221 Burwood Highway, Burwood, VIC 3125 Australia; School of Sports Science, Exercise and Health, the University of Western Australia, Crawley, WA Australia; School of Population Health, the University of Queensland, Brisbane, QLD Australia; School of Exercise and Nutrition Sciences, Deakin University, Burwood, VIC Australia; School of Public Health and Preventive Medicine, Monash University, Melbourne, VIC Australia; School of Population and Global Health, Melbourne University, Melbourne, VIC Australia; Department of Medicine, Monash University, Clayton, VIC Australia

**Keywords:** Sedentary behaviour, Built environment, Traffic, Prospective study

## Abstract

**Background:**

There has been a growing interest in environmental initiatives to reduce sedentary behaviour. A few existing studies on this topic are mostly cross-sectional, focused on the general adult population, and examining neighbourhood walkability. This study examined associations of perceived environmental attributes with change in TV viewing time over seven years among older Australian adults in the Australian Diabetes, Obesity and Lifestyle (AusDiab) study.

**Methods:**

The AusDiab study is a population-based study on diabetes and its risk factors in adults. We used the data on 1072 older adults (60+ years at baseline) collected in 2004–05 (baseline) and in 2011–12 (follow-up; 45. 4% men, mean age 67.5 years). Generalized linear modelling examined associations with 7 years change in TV viewing time of nine perceived neighbourhood-environment attributes relating to local shops, alternative routes, footpaths, parks, attractiveness, natural features, bicycle/walkway tracks, local traffic, and safety.

**Results:**

On average, participants increased their TV viewing time from 127 min/day to 137 min/day over the 7 years period. Adjusted for baseline TV viewing levels, TV viewing time at follow-up was 8% lower (95%CI: 0.85, 0.99) among those who did not perceive local traffic as a deterrent compared to those who perceived traffic as a deterrent. A trend for significant interaction between working status and the presence of a parks nearby indicated that, for those who were not working, those who reported having parks nearby had a marginal association with lower TV viewing time at follow-up than those who did not (p = 0.048).

**Conclusions:**

Overall TV viewing time increased on average by 10 minutes/day over 7 years among older Australian adults. Local traffic that makes walking difficult or unpleasant may increase older adults’ leisure-time sedentary behaviours such as TV viewing, possibly by deterring outdoor activities.

## Background

Too much sitting – as distinct from too little physical activity – is associated with adverse health outcomes [1,2], including for older adults [3]. Television (TV) viewing time is a common sedentary behaviour that occupies a large proportion of leisure-time [4]. For the general adult population, after accounting for the influence of leisure-time moderate- to vigorous-intensity physical activity, TV viewing time has been shown to be independently associated with increased cardio-metabolic risk and all-cause mortality [5-8]. In older adults, prolonged TV viewing time is also linked with poorer health outcomes [9,10] and deterioration of cognitive function [11,12].

Prolonged TV viewing time is more prevalent among older adults than other age groups [13,14]. In the United States, more than 80% of adults aged 60 years and older watch television for at least 2 hours per day, compared to 60–65% of those aged 20 to 59 years [13]. Considering rapidly aging populations in many industrialized nations and the high prevalence of sedentary behaviour in this age group, there is likely to be public health benefit from population-based strategies for reducing TV viewing time among older adults.

There has been a growing interest in environmental initiatives to promote physical activity through its potential to support large-scale, sustainable behavioural changes [15,16]. Immediate neighbourhood environments may be particularly relevant to older adults’ participation in physical activity as a result of retirement and decreased mobility [17]. Recent studies have shown attributes of neighbourhood environments, such as safety and access to destinations, to be associated with older adults’ physical activity [18,19]. Neighbourhood environmental attributes may also play a role in older adults’ sedentary behaviours, particularly TV viewing. For example, older adults who perceive their neighbourhood to be unsafe or unsupportive of physical activity may be more likely to remain indoors and watch TV. However, only a small number of studies to date have examined these relationships in general adults [20-23].

Previous research has found that high walkability (a composite measure of residential density, intersection density, land use mix, and net retail area ratio) is associated with less TV viewing time among adults [20,21]. Other environmental factors such as living in major cities and the presence of places to shops have been shown to be associated with less TV viewing time [14,24,25]. These studies suggest that broader contextual factors, including neighborhood environmental attributes, may be related to residents’ leisure-time sedentary behaviour, potentially through affecting time spent indoors. However, none of these studies have focused on older adults. Furthermore, with the exception of one recent study in adults [23], the existing studies on this topic are restricted to the examination of neighbourhood walkability only. Thus, how TV viewing time is associated with specific environmental attributes (e.g., access to shops and services, traffic, and personal safety) is not known, despite that such investigation can potentially provide more practical and policy-relevant information. Furthermore, there has been only one longitudinal study – among adults aged up to 65 years – that has found high walkability to be associated with a lower increase in TV viewing time only among non-workers [26].

The present study examined the associations of multiple neighbourhood environmental attributes with changes in TV viewing time over seven years and effect modification of the associations by gender and working status, among older Australian adults, using data from the Australian Diabetes, Obesity and Lifestyle (*AusDiab*) study.

## Methods

### Procedure and participants

The AusDiab is a national cohort study of Australian adults aged 25 years or older that initially examined the national prevalence of diabetes and related risk factors. The first round of AusDiab study was undertaken in 1999–2000 (AusDiab 1), the second round in 2004–2005 (AusDiab 2), and the third round in 2011–2012 (AusDiab 3). The study sample was drawn from private dwellings within 42 clusters of Census Collection Districts (CCD, a geographic unit defined by the Australian Bureau of Statistics with an average of 225 dwellings each). Six CCD clusters were randomly selected from each of seven Australian states and territory. Detailed study methods and attributes of participants in AusDiab 1 [27] and AusDiab 2 [28,29] have been previously reported.

The present study utilized data collected in AusDiab 2 (baseline) and AusDiab 3 (follow-up). The study sample consisted of adults aged 60 years or older in 2004–2005, who also participated in AusDiab 3 (2011–2012; n = 1549, 62.6% of the 2004–2005 sample). Of those, 477 were excluded because of the change in residence during the study period (n = 311) and missing data for relevant variables (n = 166). The final study sample size was 1072 (45.4% men). Compared to those who were excluded (n = 477), participants who were retained in the final sample were more likely to be married (p < 0.05), and have smaller waist circumference (p < 0.05). However, these two groups did not differ in age, gender proportion, educational attainment, household income, work status, change in mobility, TV viewing time, and leisure-time physical activity (LTPA). The study was approved by the Ethics committees of the International Diabetes Institute and Alfred Hospital, and written informed consent was obtained from all participants.

### Measures

#### TV viewing time

In both 2004–2005 and 2011–2012, participants reported total time spent watching TV or video/DVD on weekdays and weekends during the past week. This measure has been shown to have acceptable level of test-retest reliability (intraclass correlation = 0.82) and criterion validity (Spearman rank-order correlation with a 3-day log = 0.3) among adults [30]. The sum of weekday and weekend day TV viewing time was calculated, and then divided by seven to determine daily TV viewing time (min/day).

#### Perceived attributes of neighbourhood environments

Neighbourhood environment attributes were measured in 2004–2005 using the following nine items representing selected environmental attributes identified in previous studies of neighbourhood walkability [31,32]: presence of shops within walking distance; presence of many alternative routes; presence of footpaths on local streets; presence of parks nearby; presence of bicycle or walkway tracks nearby; attractiveness of neighbourhood; presence of pleasant natural features nearby; local traffic making it difficult to walk; and feeling safe to walk during the day. A neighbourhood or local area was defined as the area within a 10- to 15-minute walk from home. Each item was assessed on a Likert scale ranging from 1 (strongly disagree) to 4 (strongly agree). Responses were dichotomized into “strongly agree (4)” versus the other three response options [strongly disagree (1); disagree (2); agree (3)]. For the negatively worded item of the local traffic, it was dichotomized into “strongly disagree (1)” versus the other three response options. The reasons for this categorization were twofold. First, the individual items were heavily skewed with many participants agreeing strongly with the statements (40 to 85%). Second, we considered that those with strong positive or negative views on their neighbourhood environment may be more likely to alter their behaviours in line with these perceptions. We combined the “strongly agree” and “agree” response options and found no change to the findings (because of small cell sizes, we were unable to do this for all items).

#### Potential confounding variables

In 2004–2005, the following socio-demographic attributes were assessed using an interviewer-administered questionnaire: gender, age, marital status (currently married or de facto; single), educational attainment (university or further education; high school or less), household income (<AU$32,200; ≥AU$32,200 per annum), and working status (working full time or part time; not working). Mobility impairment was assessed at baseline and follow-up with a single item, asking participants to identify whether or not they had any problems in walking 500 m. Since the change in participants’ mobility status may influence their sedentary behaviour patterns, the change in mobility were determined (having problem at baseline and follow-up, having problem at follow-up only, having problem at baseline only, no problem at baseline and follow-up).

In 2004–2005 and 2011–2012, LTPA was assessed using the Active Australia Survey, a validated and reliable questionnaire [33]. Total LTPA was calculated as the sum of the time spent walking (continuous for 10 min or more), performing moderate-intensity physical activity, and double the time spent in vigorous-intensity physical activity [33]. Waist circumference was measured twice by trained staff both in 2004–2005 and 2011–2012. The mean of the two measures was calculated.

### Statistical analyses

Paired t-tests were first utilized to compare mean TV viewing time (square root transformed due to positively skewed distributions) between 2004–2005 and 2011–2012. Environmental attributes associated with TV time change were examined by modelling TV viewing time in 2011–2012, adjusting for TV viewing time in 2004–2005. This approach is equivalent to modelling change in TV viewing time and controls for regression to the mean [26]. Generalized linear modelling (GLM) with a gamma distribution and a log link was used to examine the main effects of perceived neighbourhood attributes at baseline on TV viewing time at follow-up. Perceived environmental attributes were examined individually (single-item models) and simultaneously (multiple-item model). The latter model aimed to identify relative contributions of each environmental attribute on TV viewing time. Results of each GLM model are reported as antilogarithms of the regression coefficients (Exp(b) and their respective 95%CI). They show the proportional increase (for values > 1) or decrease (for values < 1) in TV viewing time at follow-up for those who strongly agreed with each statement, compared to the reference group (those who strongly disagreed, disagreed, and agreed with the statement). For the item on local traffic, the regression coefficient was for those who strongly disagreed with the item. Interaction effects of gender, age, change in mobility, and working status with each perceived neighbourhood attribute on TV viewing time were then estimated. Stratified analysis was conducted for a significant interaction. All analyses adjusted for baseline TV viewing time, gender, age, marital status, educational attainment, household income, working status, mobility, LTPA, and waist circumference. The study had a multistage sampling design, but there was negligible clustering in TV viewing time in 2011–2012 at the level of CCD clusters (interclass correlation coefficient = 0.0063). Thus, analyses did not correct for clustering.

## Results

### Sample characteristics

Table 1 presents baseline socio-demographic, behavioural, and health-related characteristics of participants in the study. The majority of participants was women, married, not working, and had no tertiary education. Mean TV viewing time increased from 127.5 min/day to 137.5 min/day over the seven years from 2004–2005 and 2011–2012 (p < 0.001).

**Table 1:** **Characteristics of study participants in 2004–2005 (N = 1072)**

	**% or mean (SD)**
Gender (% men)	45.4
Age	67.5 (6.0)
Marital status (% married or defacto)	77.2
Educational attainment (% with university or further education)	313
Household Income (%)	
< $32,200 p.a.	51.0
≥ $32,200 p.a.	45.4
Refusing answer or missing	3.6
Work status (% non-working)	72.6
Mobility^a^ (%)	82.2
Having problem at baseline and follow-up	155 (14.5)
Having problem at follow-up only	182 (17.0)
Having problem at baseline only	46 (4.3)
No problem at baseline and follow-up	681 (63.5)
Missing	8 (0.7)
TV viewing time (min/day)	127.5 (80.2)
LTPA (min/day)	43.2 (45.5)
Waist circumference (cm)	93.7 (12.9)

All variables were from baseline except for mobility.

^a^Having problem for walking 500 m.

### Associations of 2004–2005 environmental attributes with TV viewing time in 2011–2012

Table 2 shows the results of regression analyses. Adjusting for 2004–2005 TV viewing, those who strongly disagreed with the statement that local traffic made it difficult/unpleasant to walk had a 7.0% lower (Exp(b) = 0.93; 95%CI: 0.87, 0.99) TV viewing time in 2011–2011, compared to those in the reference category. Since analysis adjusted for 2004–2005 TV viewing time (supposing baseline TV time equal), the regression coefficient can be interpreted to be the difference in TV viewing time change between the groups who differed in local traffic perception. This regression coefficient remained statistically significant in multiple-item model, which adjusted for the other environmental attributes (Exp(b) = 0.92; 95%CI: 0.85, 0.99). The other environmental attributes were not significantly associated with follow-up TV viewing time in both single-item and multiple-item models.

**Table 2:** **Regression coefficients (95%CI) for the associations of TV viewing time in 2011–2012 with perceived environmental attributes adjusting for TV viewing time in 2004–2005 (N = 1072)**

**Perceived environmental attribute**	**% of strongly agree**	**Single-item models** ^**a**^		**Multiple-item model** ^**b**^	
There are many shops to buy things within easy walking distance of my home	38.4	0.97	0.90-1.03	0.96	0.89-1.03
There are many alternative routes for getting from place to place when walking in my area	53.5	0.98	0.91-1.04	1.01	0.93-1.09
There are footpaths on all of the streets in my local area	55.4	1.01	0.95-1.08	1.04	0.96-1.12
There is a park or nature reserve in my local area that is easy to get to	73.8	0.96	0.89-1.03	0.98	0.90-1.07
There are bicycle or walkway tracks in my local area that are easy to get to	50.7	0.96	0.90-1.03	0.96	0.88-1.03
My local neighbourhood is attractive	71.4	1.04	0.97-1.12	1.07	0.98-1.16
There are pleasant natural features in my local area	63.0	1.00	0.93-1.07	1.00	0.93-1.09
There is so much local traffic along most nearby streets that make it difficult/unpleasant to walk†	48.4	0.93	0.87-0.99*	0.92	0.85-0.99*
I feel safe walking in my local area during the day	84.6	1.01	0.92-1.11	1.03	0.93-1.14

*p < 0.05.

Generalized linear model (specifying a gamma distribution and using a log link) was used.

^a^Single-item models examined each environmental attribute individually and adjusted for gender, age, marital status, education attainment, household income, work status, change in mobility from baseline to follow-up, waist circumference, TV viewing time, and LTPA at baseline.

^b^Multiple-item model examined all environmental attributes simultaneously and adjusted for all covariates used in single-item models.

† For the negatively worded item ‘so much local traffic along most nearby streets’, the expected proportional change in TV viewing time is for strong disagreement with the statement.

No statistically significant interactions were found for gender, age, change in mobility, and working status on perceived environmental attributes with TV viewing time. A trend for significant interaction was observed between working status and the presence of a park or nature reserve nearby (p = 0.100). Stratified analyses indicated that among non-working participants, those who strongly agreed with the presence of parks nearby had a 8.8% lower TV viewing time (Exp(b) = 0.91; 95%CI: 0.83, 0.99) in 2011–2012 than those who did not, which was statistically significant (p = 0.048). For participants who were working, the perceived presence of a local park was not associated with TV viewing time at follow-up (p = 0.595).

## Discussion

This is the first longitudinal study to examine the potential influence of multiple neighbourhood environment attributes on TV viewing time change among older adults. On average, daily TV viewing increased by 10 minutes during the seven years of follow-up. This may appear modest, however, 36% of participants increased their TV viewing by 30 minutes per day. Given that many participants in the current study were already “heavy TV viewers” (averaged more than two hours a day at baseline), any further increases are a public health concern. Some previous studies have examined the relationships of neighbourhood walkability with sedentary behaviour in adults aged 65 years or younger [20-22,26]. The present study expands on these findings by focusing specifically on older adults, using a longitudinal study design, and examining a range of specific environmental attributes. After controlling for LTPA, waist circumference, mobility, and socio-demographic variables, older adults who did not perceive local traffic as a deterrent to walking at all reported significantly lower TV viewing time increase at the seven-year follow-up, compared to those who found some issues with local traffic.

Previous studies have shown that various aspects of local traffic (e.g., noise, speed, inadequate signal time, or aggressive behaviour of other road users) are associated with the fear of moving outside [34,35] and with neighbourhood walking among older adults [36,37]. It is possible that older adults would be more sensitized to ‘environmental threats’ from local traffic than younger adults due to a decline in their functional capacity (e.g., strength, balance, or sensory function). Local traffic that discourages older adults from going out may increase the likelihood of them spending more time indoors engaging in sedentary behaviour such as TV viewing.

We also found that among non-working participants, those who reported having parks nearby had a marginal association with lower TV viewing time at follow-up. The present finding is consistent with those of a previous prospective study, which found low neighbourhood walkability to be associated with TV viewing time increase only among non-working adults [26]. Regardless of age, non-working adults may be likely to spend more time at home and within their neighbourhood and have more discretionary time, compared to working adults. In such circumstances, their behaviours might depend more on the surrounding neighbourhood environments than would be the case for those who are working. Considering that TV viewing time better reflects total sedentary time in non-working adults than working adults [38], improving perceptions for access to neighbourhood parks and recreational facilities might be effective for reducing indoor sedentary behaviour among non-working older adults. Future research with objective measures of park access and quasi-experimental research including ‘natural experiments’ where people are exposed to environmental changes are required to determine whether availability of neighbourhood parks can prevent an increase in TV viewing time.

The present study found no significant associations between the other environmental attributes examined (shops within walking distance, many alternative routes, the presence of footpaths, bicycle or walkway tracks nearby, attractive neighbourhood, pleasant natural features nearby, feeling safe to walk during the day) and TV viewing time at 7-year follow-up. Some of these environmental characteristics may be favourably related to older adults’ walking, but do not appear to be related to their TV viewing levels over time. It is important to consider that TV viewing time and walking are independent behaviours that can have quite different determinants [30]. It is possible that the broader neighbourhood environment (e.g., destinations, street connectivity) is relevant for those who can walk a longer distance, but an environment in close vicinity to home may be more relevant to whether older adults spend a longer time at home. Further studies exploring immediate environmental attributes close to home may be needed to identify environmental and policy initiatives to promote active living among older adults.

The major strengths of the study were its prospective design, a large sample of older adults recruited from diverse settings (urban, suburban, regional) across Australia, and examination of a broad range of perceived neighbourhood environmental attributes. Several limitations need to be considered in interpreting the present findings. The use of self-reported measures for TV viewing time and neighbourhood environmental attributes could be subject to recall error and social desirability bias. There may be some environmental changes between baseline and follow-up, which may affect change of TV viewing behaviour. Indications that loss to follow-up was not completely at random may have resulted in selection bias, which negatively affects both internal and external validities of current findings. We used baseline environmental perceptions as exposure, but changes in perceptions, which may be caused by functional declines, may affect TV viewing behaviour. The association observed between perceptions of local traffic and TV viewing time may be due to unmeasured variables, such as neighbourhood socio-economic status. Furthermore, this study only measured TV viewing, not other common recreational sedentary behaviours among older adults, such as reading, crafting, and sedentary social activities. Though TV viewing time may be reflective of a broader sedentary lifestyle, it is likely to be associated with unique correlates and health outcomes. For example, increasing TV viewing with ageing may reflect increasing social isolation and could be detrimental to older adults’ quality of life and well-being [39]. Future studies should measure a broader range of recreational sedentary behaviours and assess social and mental health correlates and outcomes.

## Conclusions

In summary, overall TV viewing time increased on average by 10 minutes/day over 7 years among older Australian adults. TV viewing time, a common leisure-time sedentary behaviour among older adults, may change differently by perceptions of local traffic conditions and potentially by other neighbourhood attributes, for which traffic could be a marker (e.g., social norm). However, local traffic involves multiple factors, such as vehicle speed, volume, noise, fume, and traffic-related infrastructure. Further studies examining even more specific environmental attributes with objective behavioural measures are needed to better understand what environmental attributes should be given specific focus in order to reduce older adults’ sedentary behaviour.

## Abbreviations

AusDiab study: Australian Diabetes: Obesity and Lifestyle study
TV: Television
LTPA: Leisure-time physical activity
GLM: Generalized linear modelling
